# Supplementary material for: PLM-interact: extending protein language models to predict protein-protein interactions
Source: Nat Commun. 2025 Oct 27;16:9012. doi: 10.1038/s41467-025-64512-w (PMC12559430; doi:10.1038/s41467-025-64512-w)
Supplement: Supplementary file 1 — Supplementary Information [file 41467_2025_64512_MOESM1_ESM.pdf]

Supplementary Figures

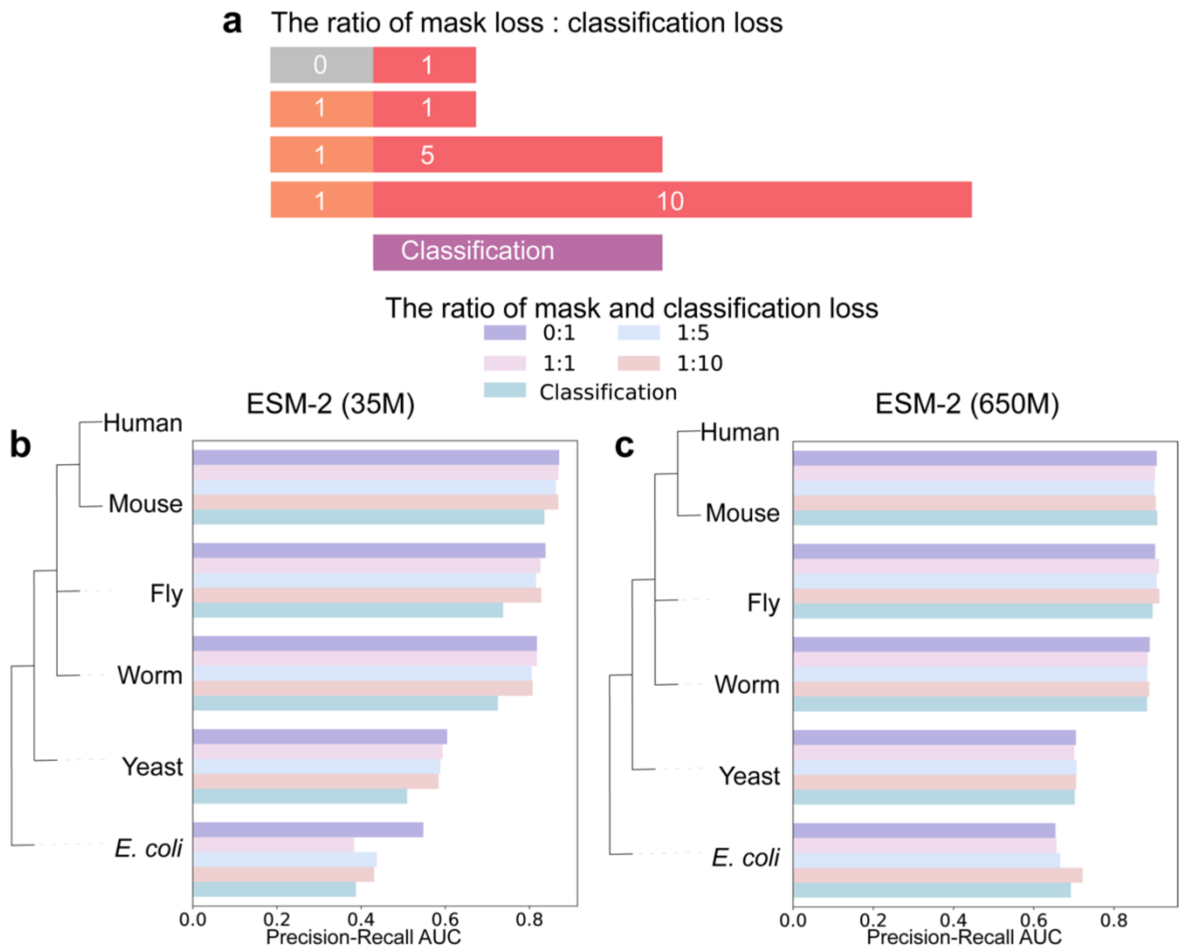

Supplementary Figure 1. Performance of PLM-interact on protein-protein interaction (PPI) prediction across five test species (mouse, fly, worm, yeast and *E. coli*). a. Bar plots showing the different ratios between mask and classification losses. **b**, **c**. The benchmarking results of PLM-interact trained with different mask-to-classification loss ratios. **b** shows AUPR values from models trained with 35M ESM-2, and **c** shows AUPR values from models trained with 650M ESM-2. The x-axis shows AUPR values, and the y-axis is aligned with the taxonomy tree of the test host species. Source data are provided as a Source Data file.

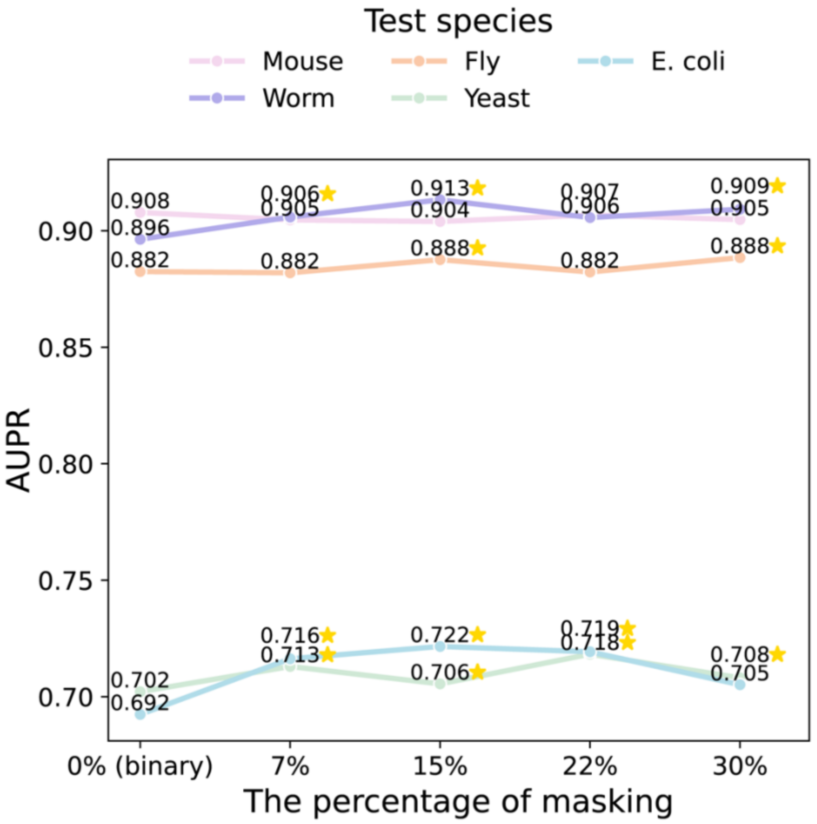

Supplementary Figure 2. The AUPR values of the PLM-interact training with the different masking percentages on each test species. The x-axis represents the percentage of masking; the y-axis represents the AUPR value of each trained model on each test species. The gold star indicates that the masking model has a significant improvement over the binary model (without masking), and p-value < 0.05 with McNemar's Test. Source data are provided as a Source Data file.

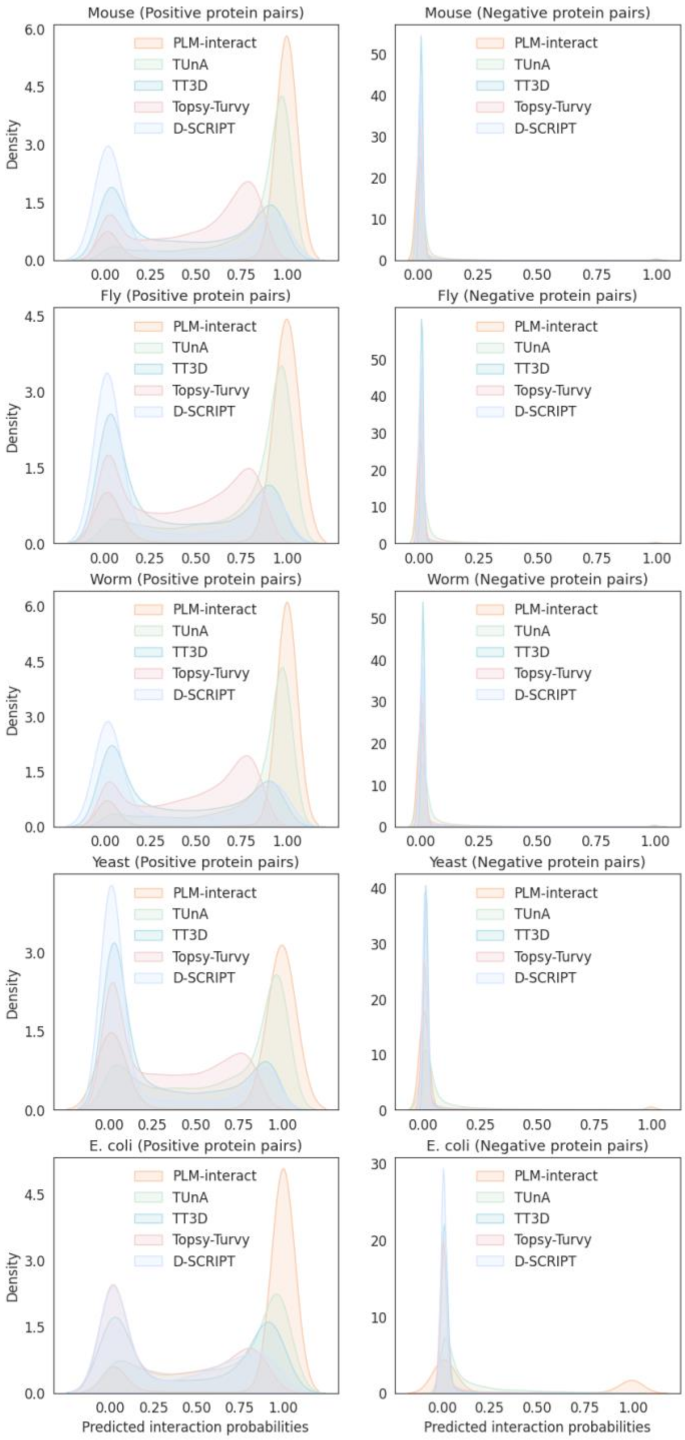

Supplementary Figure 3. Distribution of predicted interaction probabilities of positive and negative protein pairs for five models: PLM-interact, TUnA, TT3D, Topsy-Turvy and D-SCRIPT. PLM-interact outperforms other models by identifying the most true positive pairs (predicted interaction probability > 0.5) and true negative pairs (predicted interaction probability < 0.5), demonstrating that PLM-interact achieves the best performance, except for negative pairs of *E. coli*. Source data are provided as a Source Data file.

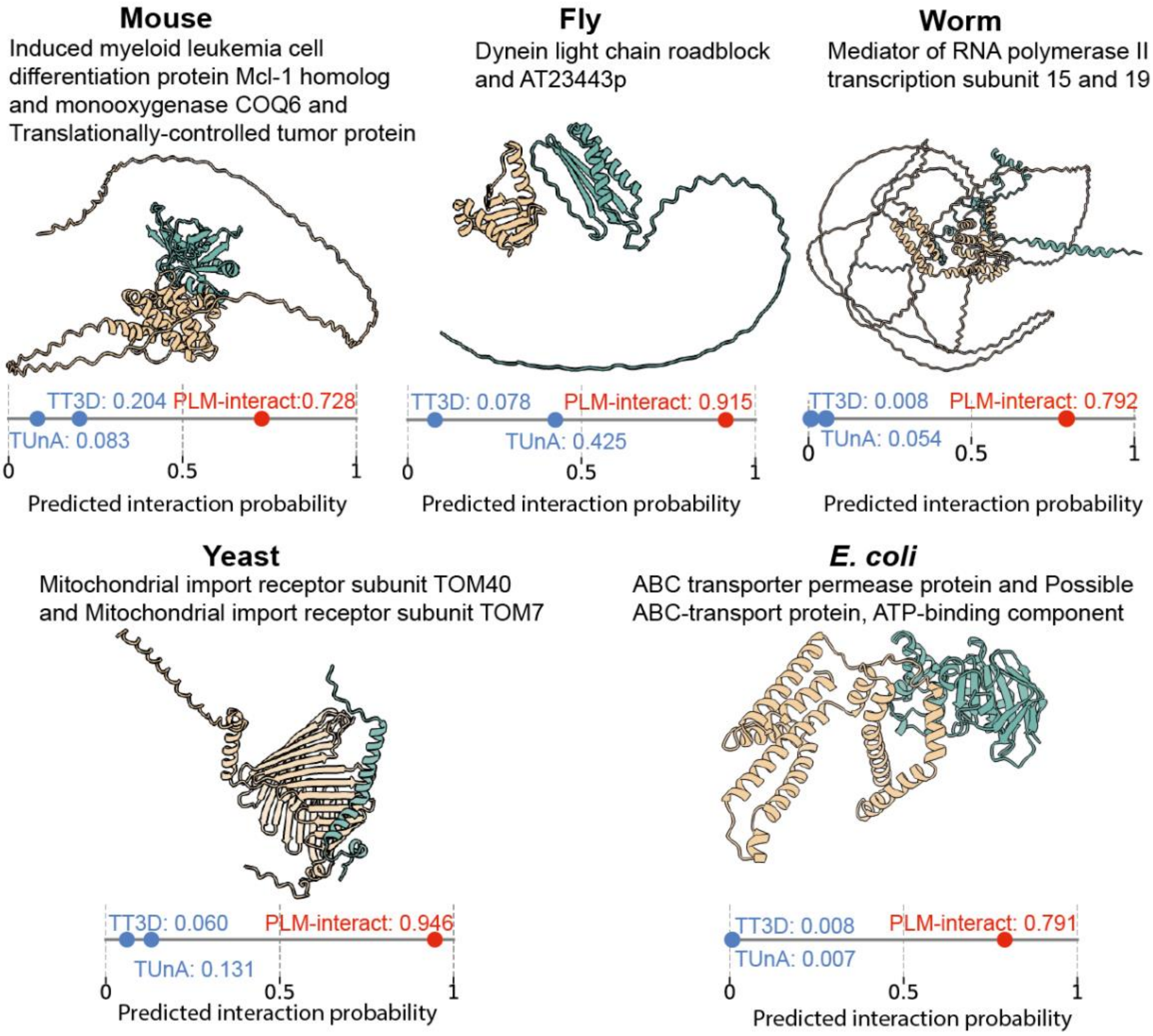

Supplementary Figure 4. Protein-protein interaction (PPI) example for each species that is predicted correctly by PLM-interact but not by TUnA and TT3D. Protein-protein structures are predicted by AlphaFold3[1] and visualised with ChimeraX[2]. Both models' prediction interaction probabilities range between 0 and 1. A predicted interaction probability >0.5, is predicted as a positive PPI, while <0.5 is a negative pair. Interacting proteins are shown from left (yellow) to right (green), respectively. For information about these PPIs, see **Figure 3**. Source data are provided as a Source Data file.

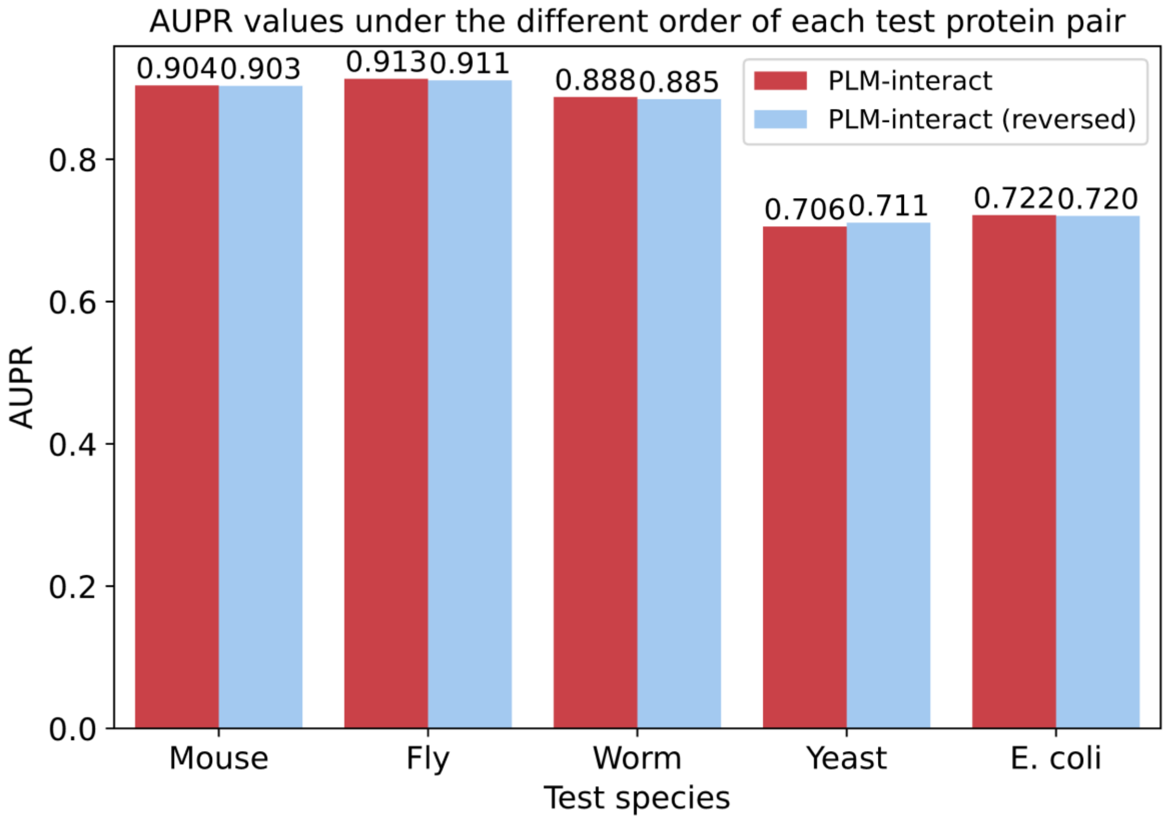

Supplementary Figure 5. The AUPR bar plot shows the impact of the original and reversed test protein pairs on PLM-interact performance. The x-axis represents five test species, and the y-axis represents AUPR values. The AUPR values are obtained based on inference using the original and reversed order of the test protein pairs from each species. Source data are provided as a Source Data file.

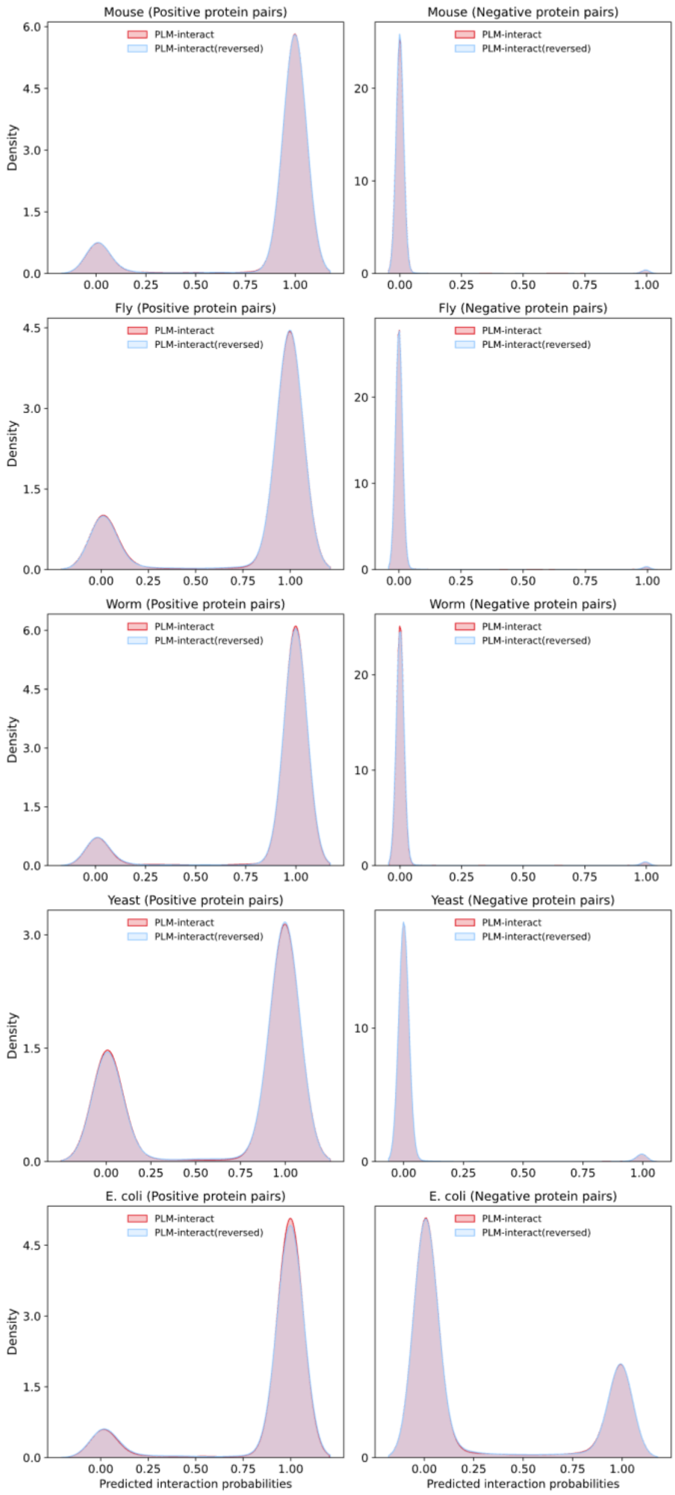

Supplementary Figure 6. The distribution of predicted interaction probabilities between the original and reversed order of each test protein pair from mouse, fly, worm, yeast and *E. coli*, respectively. The predicted interaction probabilities between the original and reversed order in each test protein pair exhibits almost identical distributions. Source data are provided as a Source Data file.

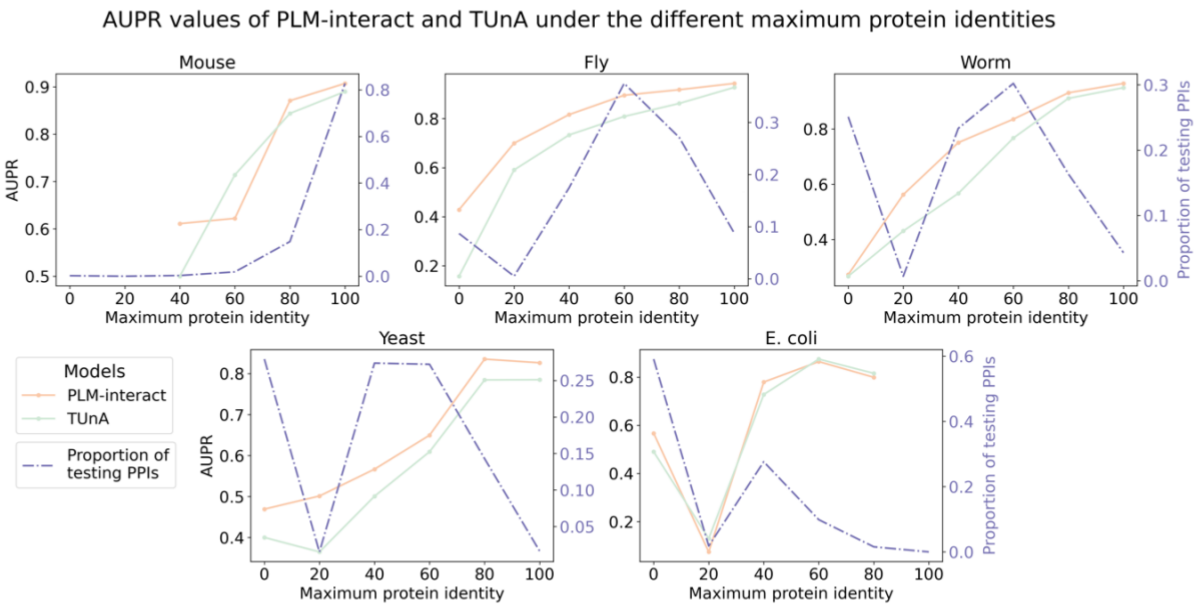

Supplementary Figure 7. The AUPR performance of PLM-interact and TUnA under different levels of protein identities between training and test datasets. The x-axis represents the identity of protein sequence between training and test proteins. For each panel, the left y-axis represents AUPR values, and the right y-axis represents the proportion of the test protein pairs under the corresponding protein identities (x-axis). The figure shows AUPR values of PLM-interact and TUnA on test protein pairs of mouse, fly, worm, yeast and *E. coli*, respectively. Their performance improves as protein

identity increases, with PLM-interact consistently outperforming TUnA. Source data are provided as a Source Data file.

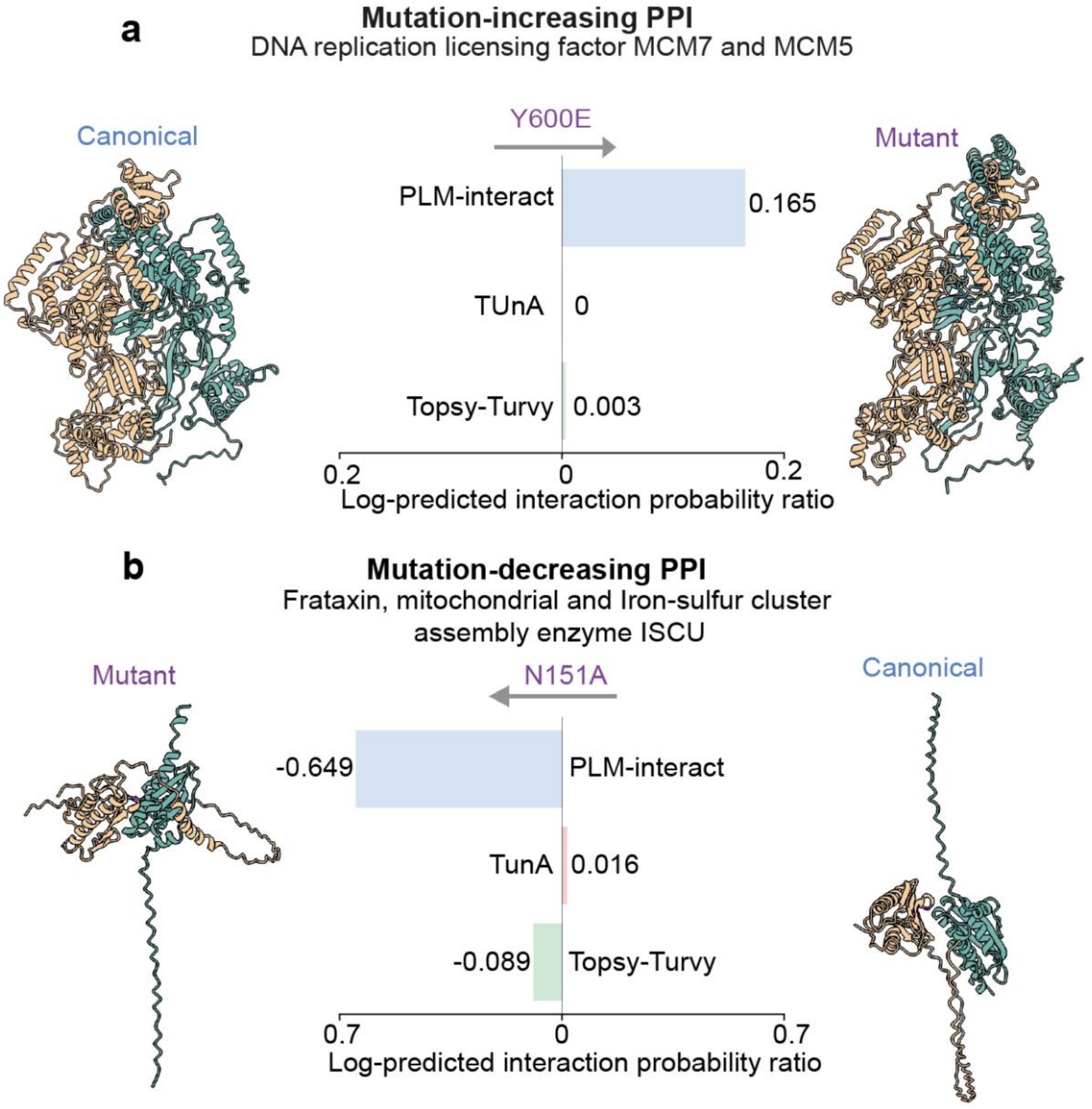

Supplementary Figure 8. Demonstration of PLM-interact detecting changes in human protein-protein interactions (PPIs) associated with mutations. **a** Shows an example of a mutation causing an increase in binding affinity, while **b** shows a mutation causing a decrease in binding affinity. These PPI structures are predicted using AlphaFold3[1] and visualised with ChimeraX[2]; here, the mutated amino acids are highlighted in purple. A log-predicted interaction probabilities ratio between the mutant and canonical pairs above 0 indicates a mutation-increasing PPI, while a ratio below 0 indicates a mutation-decreasing PPI. AlphaFold3's ipTM and pTM scores shown in **Supplementary Table 3** give the structure prediction confidence. Interacting protein structures are shown from left (yellow) to right

(green). See **Figure 6** for information about these protein pairs. Source data are provided as a Source Data file.

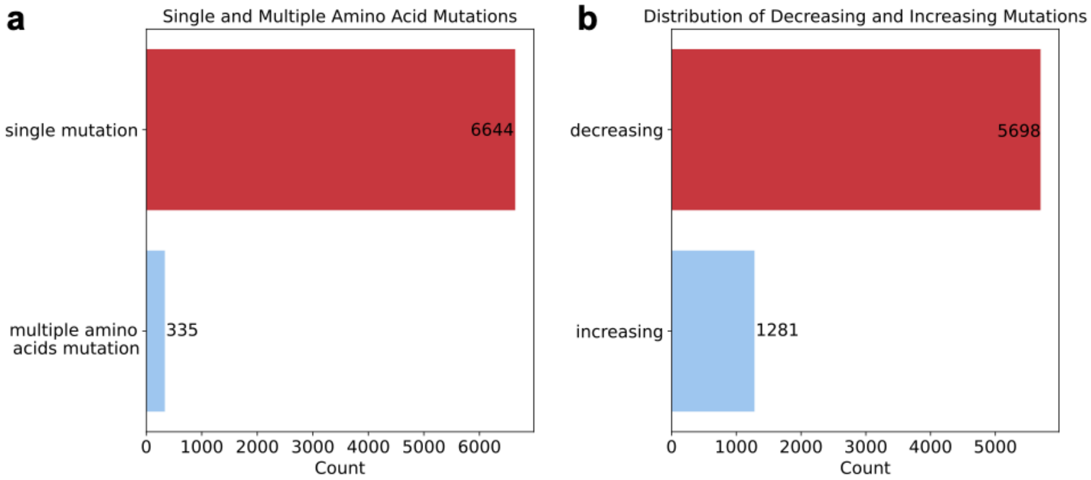

Supplementary Figure 9. Counts of mutant types in the mutation dataset. a. The number of mutation samples with single versus multiple amino acid substitutions; **b**. The number of mutation samples with mutations increasing versus decreasing interactions. Source data are provided as a Source Data file.

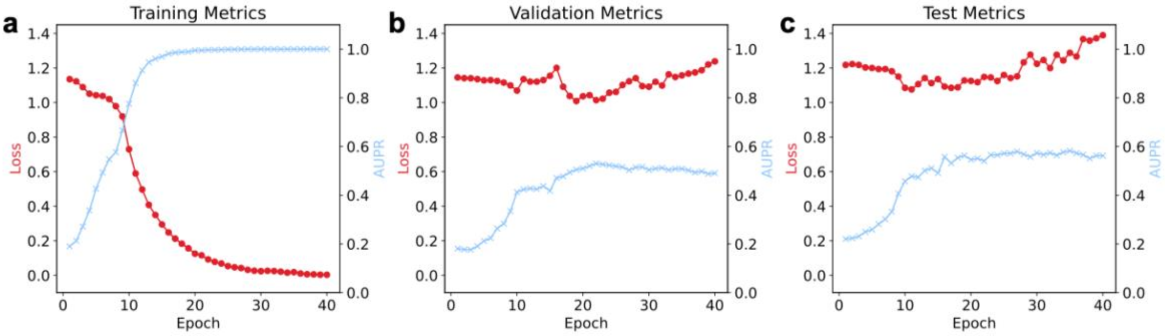

Supplementary Figure 10. The loss and AUPR values with the training epochs for training, validation and test datasets. We fine-tune all layers of PLM-interact for mutation effect prediction. The model's losses and performance during training, validation and testing are shown in panels **a**, **b** and **c**, respectively. In panel **b**, the validation loss reaches a minimum at epoch 19, after which further training increases the validation loss. Therefore, epoch 19 is chosen as the final model for mutation effect prediction. Source data are provided as a Source Data file.

Tables

Supplementary Table 1. This table shows the GPU hours (GPUhs) of different models. PLM-interact has the two kinds of training strategies: masking language modelling and binary classification, binary classification only. The ratios in this table present the different weights between mask loss and classification loss, binary indicates the binary classification task without masking.

**Models/GPUhs**	**650M**	**35M**
0:1	522.4	238.2
1:1	514.3	299.1
1:5	508.4	238.1
1:10	496.9	240.4
Binary	355.8	130.9

Supplementary Table 2. The table of ipTM and pTM scores of predicted 3D structures from Chai-1 and AlphaFold3 for each test species in **Figure 3** and **Supplementary Figure 4**. The ipTM and pTM are the confidence scores of the prediction structure. For details about both scores, refer to the AlphaFold3 paper[1].

	**Chai-1**		**AlphaFold3**	
	**ipTM**	**pTM**	**ipTM**	**pTM**
Mouse	0.15	0.43	0.17	0.37
Fly	0.13	0.43	0.09	0.36
Worm	0.11	0.24	0.11	0.21
Yeast	0.74	0.78	0.66	0.65
E. coli	0.79	0.88	0.82	0.78

Supplementary Table 3. The table of ipTM and pTM scores of predicted 3D structures from Chai-1 and AlphaFold3 for two mutation protein-protein interaction (PPI) examples in **Figure 6** and **Supplementary Figure 8**. The ipTM and pTM are the confidence scores of the prediction structure. For details about both scores, refer to the AlphaFold3 paper[1].

		**Increasing (P33993 - P33992)**		**Decreasing (Q16595-Q9H1K1)**	
		**ipTM**	**pTM**	**ipTM**	**pTM**
**Chai-1**	**Canonical**	0.64	0.74	0.12	0.45

	**Mutant**	0.65	0.74	0.15	0.46
**AlphaFold3**	**Canonical**	0.62	0.68	0.15	0.38
	**Mutant**	0.48	0.60	0.15	0.38

Supplementary Table 4. The table summarises the features, architectures, references and code links of state-of-the-art protein-protein interaction (PPI) models evaluated in this study.

	**Protein features**	**Model architecture**	**Source**	**Tool**
TUnA	Pre-trained PLM Embeddings	Spectral-normalized Neural Gaussian Process	Young, S. et al. 2024	https://github.com/Wang-lab-UCSD/TUnA
TT3D	Pre-trained Bepler & Berger PLM Embeddings and one-hot encoding of the Foldseek 3Di	Convolutional neural network	Sledzieski, S. et al. 2023	https://github.com/samsledje/D-SCRIPT
D-SCRIPT	Pre-trained Bepler & Berger PLM embeddings	Convolutional neural network	Sledzieski, S. et al. 2021	http://dscript.csail.mit.edu
Topsy-Turvy	Pre-trained Bepler & Berger PLM embeddings and network structure	Intergrade D-SCRIPT and a network-based model GLIDE	Singh, R. et al. 2022	https://topsyturvy.csail.mit.edu
PIPR	Pre-trained amino acid embeddings using Skip-Gram model	Siamese residual RCNN	Chen, M. et al. 2019	https://github.com/muhaochen/seq_ppi
DeepPPI	One hot encoding amino acids	Fully connected model architecture	Richoux et al., 2019	https://gitlab.univ-nantes.fr/richoux-f/DeepPPI
STEP	Pre-trained PLM ProtBERT embeddings	Siamese neural network	Madan, S. et al. 2022	https://github.com/SCAI-

				BIO/STEP/tree/main
LSTM-PHV	word2vec	LSTM and Siamese model	Tsukiyama et al.2021	http://kurata35.bio.kyutech.ac.jp/LSTM-PHV.
InterSPPI	doc2vec	Random Forest classifier	Yang et al. 2020	http://zzdlab.com/InterSPPI/

Supplementary References

1. Abramson, J. *et al.* Accurate structure prediction of biomolecular interactions with AlphaFold 3. *Nature* **630**, 493–500 (2024).

2. Pettersen, E. F. *et al.* UCSF ChimeraX: Structure visualization for researchers, educators, and developers. *Protein Science* **30**, 70–82 (2021).
