# Supplementary material for: PLM-interact: extending protein language models to predict protein-protein interactions
Source: Nat Commun. 2025 Oct 27;16:9012. doi: 10.1038/s41467-025-64512-w (PMC12559430; doi:10.1038/s41467-025-64512-w)
Supplement: Supplementary file 2 — Reporting Summary [file 41467_2025_64512_MOESM2_ESM.pdf]

nature portfolio

Corresponding author(s): Craig Macdonald, David L Robertson, Ke Yuan

Last updated by author(s): Aug 8, 2025

Reporting Summary

Nature Portfolio wishes to improve the reproducibility of the work that we publish. This form provides structure for consistency and transparency in reporting. For further information on Nature Portfolio policies, see our Editorial Policies and the Editorial Policy Checklist.

Statistics

For all statistical analyses, confirm that the following items are present in the figure legend, table legend, main text, or Methods section.

n/a	Confirmed	
☐	☒	The exact sample size (*n*) for each experimental group/condition, given as a discrete number and unit of measurement
☒	☐	A statement on whether measurements were taken from distinct samples or whether the same sample was measured repeatedly
☐	☒	The statistical test(s) used AND whether they are one- or two-sided<br>*Only common tests should be described solely by name; describe more complex techniques in the Methods section.*
☒	☐	A description of all covariates tested
☒	☐	A description of any assumptions or corrections, such as tests of normality and adjustment for multiple comparisons
☐	☒	A full description of the statistical parameters including central tendency (e.g. means) or other basic estimates (e.g. regression coefficient) AND variation (e.g. standard deviation) or associated estimates of uncertainty (e.g. confidence intervals)
☐	☒	For null hypothesis testing, the test statistic (e.g. *F*, *t*, *r*) with confidence intervals, effect sizes, degrees of freedom and *P* value noted<br>*Give P values as exact values whenever suitable.*
☒	☐	For Bayesian analysis, information on the choice of priors and Markov chain Monte Carlo settings
☒	☐	For hierarchical and complex designs, identification of the appropriate level for tests and full reporting of outcomes
☒	☐	Estimates of effect sizes (e.g. Cohen's *d*, Pearson's *r*), indicating how they were calculated

Our web collection on statistics for biologists contains articles on many of the points above.

Software and code

Policy information about availability of computer code

Data collection

The benchmarking human protein-protein interactions (PPIs) are produced by Sledzieski et al. retrieving from the protein-protein networks STRING V11(https://version-11-0.string-db.org/). PPIs and protein sequences are publicly available at https://d-script.readthedocs.io/en/stable/data.html. The gold standard human dataset is sourced from HIPPIE v2.3 and available at https://doi.org/10.6084/m9.figshare.21591618.v3. The benchmarking virus-human PPIs are sourced from HPIDB 3.0 and can be downloaded at http://kurata35.bio.kyutech.ac.jp/LSTM-PHV/download_page.

The training, validation and test datasets for the mutation effect classification task are sourced from the IntAct molecular interaction database (https://ftp.ebi.ac.uk/pub/databases/intact/current/various/mutations.tsv) and are available at https://huggingface.co/datasets/danliu1226/Mutation_effect_dataset.

We created a human PPI dataset retrieved from STRING V12 (https://stringdb-downloads.org/download/protein.physical.links.v12.0.txt.gz). To remove the PPI samples with high protein identities, we used MMseq2 Release 13-45111 to obtain the protein identities between each protein pair.

3D complex structures of human-virus PPIs are from the public dataset HVIDB (http://zzdlab.com/hvidb/download.php, updated on 25 June 2020). Protein sequences are retrieved from UniProt https://www.uniprot.org/, access on 10 Oct 2023.

Code, python version, packages and instructions can be found in the GitHub repository: https://github.com/liudan111/PLM-interact
All generated model predictions and preprocessed datasets are available at Source Data and https://huggingface.co/danliu1226.

Data analysis

The Code in this study is publicly available and has been deposited in GitHub at https://github.com/liudan111/PLM-interact. The specific version of the code associated with this publication is archived in Zenodo and is accessible via https://doi.org/10.5281/zenodo.16643326. Trained model checkpoints and datasets used in this study are available at https://huggingface.co/danliu1226.

For manuscripts utilizing custom algorithms or software that are central to the research but not yet described in published literature, software must be made available to editors and reviewers. We strongly encourage code deposition in a community repository (e.g. GitHub). See the Nature Portfolio guidelines for submitting code & software for further information.

Data

Policy information about availability of data

All manuscripts must include a data availability statement. This statement should provide the following information, where applicable:

- Accession codes, unique identifiers, or web links for publicly available datasets
- A description of any restrictions on data availability
- For clinical datasets or third party data, please ensure that the statement adheres to our policy

The cross-species benchmarking dataset created by Sledzieski et al. is available at https://d-script.readthedocs.io/en/stable/data.html. The gold standard human dataset created by Bernett et al. is available at https://doi.org/10.6084/m9.figshare.21591618.v3. The virus-human benchmarking PPI dataset created by Tsukiyama et al. is available at http://kurata35.bio.kyutech.ac.jp/LSTM-PHV/download_page. Protein sequences are retrieved from UniProt (https://www.uniprot.org/). The 3D complex structures of human-virus PPIs are obtained from HVIDB (http://zzdlab.com/hvidb/download.php). The STRING V12 human training data are sourced from the STRING PPI database V12 (https://stringdb-downloads.org/download/protein.physical.links.v12.0.txt.gz) and are available at https://huggingface.co/datasets/danliu1226/STRING_V12_TrainingSet. The training, validation and test datasets for the mutation effect classification task are sourced from the IntAct molecular interaction database (https://ftp.ebi.ac.uk/pub/databases/intact/current/various/mutations.tsv) and are available at https://huggingface.co/datasets/danliu1226/Mutation_effect_dataset. The data generated in this study (training, validation and test datasets used for PPI benchmarking and mutation effect prediction tasks, cross-species PPI model checkpoints; the Bernett PPI model checkpoint; mutation effect classification model checkpoint; predicted interaction probabilities for PPI models on benchmarking tasks; prediction results for mutation effect classification and PPI model evaluations under varying protein sequence identities between training and test datasets) are available at Hugging face (https://huggingface.co/danliu1226) and the Source Data file. All datasets in this study are publicly accessible without restrictions. Source data are provided with this paper.

Research involving human participants, their data, or biological material

Policy information about studies with human participants or human data. See also policy information about sex, gender (identity/presentation), and sexual orientation and race, ethnicity and racism.

Reporting on sex and gender	This study contains no human participants.
Reporting on race, ethnicity, or other socially relevant groupings	NA
Population characteristics	NA
Recruitment	NA
Ethics oversight	NA

Note that full information on the approval of the study protocol must also be provided in the manuscript.

Field-specific reporting

Please select the one below that is the best fit for your research. If you are not sure, read the appropriate sections before making your selection.

☒ Life sciences ☐ Behavioural & social sciences ☐ Ecological, evolutionary & environmental sciences

For a reference copy of the document with all sections, see nature.com/documents/nr-reporting-summary-flat.pdf

Life sciences study design

All studies must disclose on these points even when the disclosure is negative.

Sample size

The sample size is determined by the size of public databases and the preprocessing methods applied. No statistical methods are used to calculate sample size. Samples are protein-protein interactions (PPIs) collected from public databases: STRING V11, STRING V12, HIPPIE v2.3 and HPIDB 3.0 PPI. PPIs with a positive experimental score are selected as positive samples, while negative protein pairs are generated by randomly pairing proteins that are not reported to interact. The gold standard benchmarking dataset collected from HIPPIE v2.3 has a 1:1 ratio of positive to negative protein pairs; all other datasets maintain a 1:10 ratio of positive to negative pairs, reflecting that positive PPIs are significantly fewer than negative pairs in the actual PPI networks. Based on previous PPI studies, these sample sizes are sufficient for model PPI training and benchmarking.

References:

[1] Sledzieski, S., Singh, R., Cowen, L. & Berger, B. D-SCRIPT translates genome to phenome with sequence-based, structure-aware, genome-scale predictions of protein-protein interactions. Cell Systems 12, 969-982.e6 (2021).

[2] BBernett, J., Blumenthal, D. B. & List, M. Cracking the black box of deep sequence-based protein–protein interaction prediction. Briefings in

Bioinformatics 25, bbae076 (2024).
[3]Tsukiyama, S., Hasan, M. M., Fujii, S. & Kurata, H. LSTM-PHV: prediction of human-virus protein–protein interactions by LSTM with word2vec. Briefings in Bioinformatics 22, bbab228 (2021).

Data exclusions

Proteins are filtered based on a sequence length threshold to reduce GPU storage consumption. A confidence score threshold is applied to exclude low-quality PPIs. CD-HIT or MMSeq2 is used to cluster proteins for removing redundant PPIs with high protein sequence identities.
(1) Cross-species benchmarking dataset. Protein sequence lengths range from 50 to 800 amino acids. PPIs are clustered at 40% sequence identity using CD-HIT to remove the redundant PPIs.
(2) Bernett benchmarking dataset. We train PLM-interact on protein pairs with the maximum combined paired length of 2193. CD-HIT was used to remove redundant protein pairs that have more than 40% protein sequence similarity with existing training protein pairs.
(3) Mutation effect dataset. We prioritise short protein pairs (< 2201 amino acids, ~80% of the data) as training dataset while allowing the model to train on more samples.
(4) Virus-human PPI benchmarking dataset. The length of protein sequences ranges from 30 to 1,000, and CD-HIT was used to remove redundant PPIs based on a threshold of 95% identity.
(5) The human PPIs collected from STRING V12. We process the length of paired protein more than 2000. Protein sequences are clustered at 40% identity using MMSeq2, and only PPIs from the distinct clusters are chosen to eliminate redundant PPIs.

Replication

We used multiple benchmarking datasets to train and evaluate models. We have uploaded training, prediction and evaluation codes and all training, validation, test datasets used in this study to GitHub (https://github.com/liudan111/PLM-interact) and Hugging Face (https://huggingface.co/danliu1226). Additionally, to investigate our model's performance with different masking percentages during training, McNemar's test is used to assess whether the masking model is significantly better than the binary model without masking.

Randomization

As for the benchmarking tasks, training, validation, and test sets were randomly split or constructed using a standard strategy to minimise sequence similarity between the training and test datasets. In the mutation effect prediction task and the STRING V12 human PPI model training, datasets were randomly split into training, validation, and test sets. In all PPI prediction tasks, negative protein pairs were generated by randomly pairing proteins that were not reported to interact.

Blinding

N/A. Blinding was not applicable, as this is a computational study with no human subjects involved. During training, the protein pairs are randomly masked for mask language modelling. The test dataset is randomly split from the dataset for prediction.

Reporting for specific materials, systems and methods

We require information from authors about some types of materials, experimental systems and methods used in many studies. Here, indicate whether each material, system or method listed is relevant to your study. If you are not sure if a list item applies to your research, read the appropriate section before selecting a response.

Materials & experimental systems

n/a	Involved in the study
☒	☐ Antibodies
☒	☐ Eukaryotic cell lines
☒	☐ Palaeontology and archaeology
☒	☐ Animals and other organisms
☒	☐ Clinical data
☒	☐ Dual use research of concern
☒	☐ Plants

Methods

n/a	Involved in the study
☒	☐ ChIP-seq
☒	☐ Flow cytometry
☒	☐ MRI-based neuroimaging

Plants

Seed stocks

Report on the source of all seed stocks or other plant material used. If applicable, state the seed stock centre and catalogue number. If plant specimens were collected from the field, describe the collection location, date and sampling procedures.

Novel plant genotypes

Describe the methods by which all novel plant genotypes were produced. This includes those generated by transgenic approaches, gene editing, chemical/radiation-based mutagenesis and hybridization. For transgenic lines, describe the transformation method, the number of independent lines analyzed and the generation upon which experiments were performed. For gene-edited lines, describe the editor used, the endogenous sequence targeted for editing, the targeting guide RNA sequence (if applicable) and how the editor was applied.

Authentication

Describe any authentication procedures for each seed stock used or novel genotype generated. Describe any experiments used to assess the effect of a mutation and, where applicable, how potential secondary effects (e.g. second site T-DNA insertions, mosiacism, off-target gene editing) were examined.
